# Correction: Impact of self-perceived discomfort in critically ill patients on the occurrence of psychiatric symptoms in post-intensive care syndrome (PICS): A prospective observational study

**DOI:** 10.1371/journal.pone.0332509

**Published:** 2025-09-15

**Authors:** Romain Ronflé, Julie Hermitant, Christine Conti-Zolin, Laurent Lefebvre, Thibault Helbert, Aurélien Culver, Florence Molenat, Baptiste Borwell, Mohamed Boucekine, Pierre Kalfon, Marc Leone

The eighth author’s name is spelled incorrectly. The correct name is: Baptiste Borwell.
