# Supplementary figures and images for: Impact of self-perceived discomfort in critically ill patients on the occurrence of psychiatric symptoms in post-intensive care syndrome (PICS): A prospective observational study
Source: PLoS One. 2025 Jun 6;20(6):e0324099. doi: 10.1371/journal.pone.0324099 (PMC12143565; doi:10.1371/journal.pone.0324099)

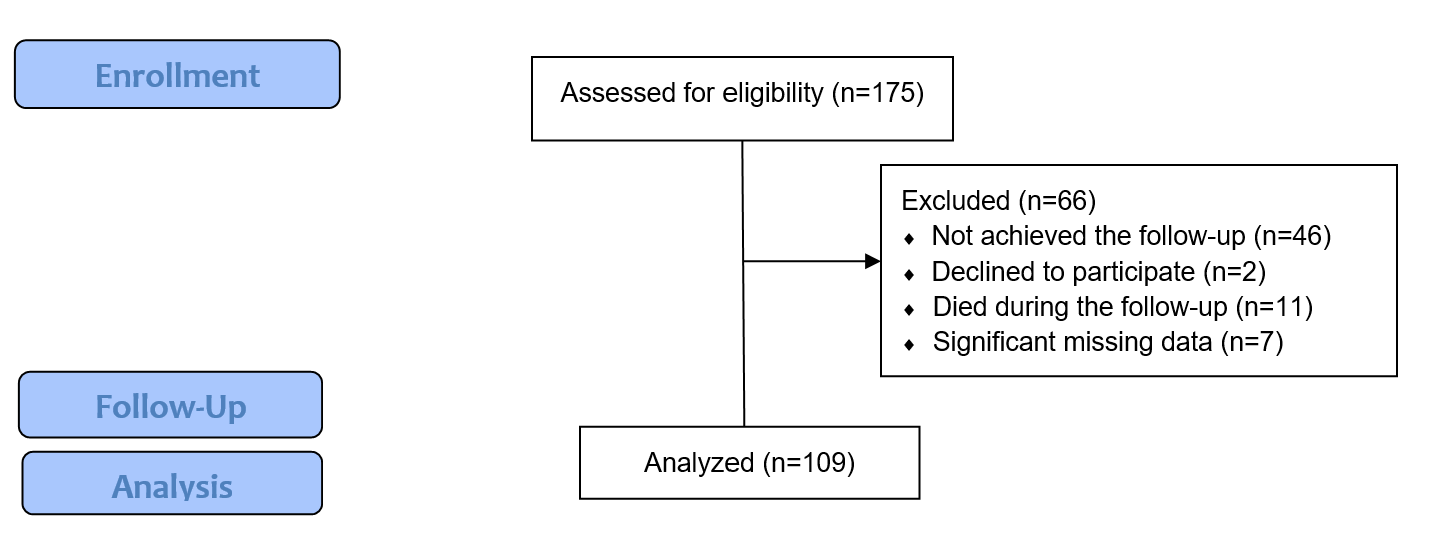

Supplement: S1 Fig — (TIF) [file pone.0324099.s001.tif]
