# Supplementary material for: Impact of self-perceived discomfort in critically ill patients on the occurrence of psychiatric symptoms in post-intensive care syndrome (PICS): A prospective observational study
Source: PLoS One. 2025 Jun 6;20(6):e0324099. doi: 10.1371/journal.pone.0324099 (PMC12143565; doi:10.1371/journal.pone.0324099)
Supplement: S1 Table — (DOCX) [file pone.0324099.s002.docx]

**Supplementary table 1. The French IPREA questionnaire for assessing self-reported discomforts perceived by the critically ill patients, original version**

1 Avez-vous souffert du ***bruit*** (alarmes, radios, sonneries de téléphone, conversations) de jour comme de nuit?

2 Avez-vous souffert de la ***lumière*** (éclairage trop important dans la chambre ou dans le couloir surtout la nuit?

3 Avez-vous souffert du ***lit*** (matelas trop dur ou trop mou, matelas à eau, tête de lit trop ou pas assez relevée, lit trop bas ou trop haut, barrières, mauvais oreillers, etc.)?

4 Avez-vous souffert du manque de ***sommeil*** par rapport à d'habitude?

5 Avez-vous souffert de la ***soif***?

6 Avez-vous souffert de la ***faim***?

7 Avez-vous souffert du ***froid***?

8 Avez-vous souffert de la ***chaleur***?

9 Avez-vous eu des ***douleurs***, même si elles étaient présentes avant l'hospitalisation, y compris les douleurs liées aux piqûres ou lors des changes ou de la toilette matinale?

10 Avez-vous souffert d'être entouré de ***tuyaux*** (pour les perfusions, les connections des électrodes fixées sur le thorax, l'oxygène dans le nez ou sur le masque, la pince pour surveiller l'oxygénation, etc.)?

11 Avez-vous été gêné par le fait que votre ***intimité*** ne soit pas suffisamment respectée (par ex. pendant la toilette matinale, les changes, l'examen par les médecins, ou les visites médicales)?

12 Avez-vous souffert d'a***ngoisse*** (peur parfois panique par exemple qu'un appareil important fonctionne mal, provoquée parfois par le déclenchement d’alarmes sonores) ou vous êtes vous senti très anxieux durant votre hospitalisation?

13 Avez-vous souffert d’***isolement*** (être seul dans votre chambre, parfois sans voir d'infirmiers ou de médecins à proximité) et sans entendre de bruit?

14 Avez-vous été gêné par la limitation des ***visites*** des membres de votre famille ou de vos amis selon les horaires de visite en vigueur dans le service?

15 Avez-vous été gêné de ne pas avoir de ***téléphone*** dans la chambre?

16 Avez-vous été gêné de n'être pas assez ***informé*** de votre état ou de ce qu'on allait vous faire, de l'évolution de votre maladie, de votre date de sortie de réanimation, des suites, que ce soit par les infirmières ou les médecins?

17 Avez-vous souffert d’une sensation de ***souffle court*** (difficulté à respirer, impression d’étouffement) ?

18 Avez-vous souffert d’une ***humeur triste*** ?

*Note: chaque item d’inconfort est en gras.*
