# Supplementary material for: Impact of self-perceived discomfort in critically ill patients on the occurrence of psychiatric symptoms in post-intensive care syndrome (PICS): A prospective observational study
Source: PLoS One. 2025 Jun 6;20(6):e0324099. doi: 10.1371/journal.pone.0324099 (PMC12143565; doi:10.1371/journal.pone.0324099)
Supplement: S2 Table — (DOCX) [file pone.0324099.s003.docx]

| Supplementary table 2. Self-reported discomforts perceived by the critically ill patients | |
| --- | --- |
| Overall discomfort score*, mean ± SD | 20 ± 14 |
| Variables | **Cohort (n=173)** |
| Noise, mean ± SD | 2.2 ± 2.7 |
| Excess of light, mean ± SD | 1.8 ± 2.5 |
| Bed-related discomfort, mean ± SD | 2.5 ± 3.3 |
| Sleep deprivation, mean ± SD | 2.8 ± 3.2 |
| Thirst, mean ± SD | 3.0 ± 3.5 |
| Hunger, mean ± SD | 1.3 ± 2.8 |
| Feeling of cold, mean ± SD | 2.2 ± 3.1 |
| Feeling of heat, mean ± SD | 0.6 ± 1.7 |
| Pain, mean ± SD | 1.9 ± 2.9 |
| Perfusion lines, etc, mean ± SD | 2.6 ± 3.0 |
| Lack of intimacy, mean ± SD | 1.9 ± 2.8 |
| Anxiety, mean ± SD | 3.8 ± 3.4 |
| Isolation, mean ± SD | 1.6 ± 2.7 |
| Limited visiting hours, mean ± SD | 0.7 ± 1.8 |
| Absence of a phone, mean ± SD | 0.5 ± 1.7 |
| Lack of information, mean ± SD | 1.2 ± 2.3 |
| Shortness of breath, mean ± SD | 3.0 ± 3.3 |
| Feeling depressed, mean ± SD | 3.0 ± 3.2 |

Values are expressed as mean ± SD, *derived from IPREA questionnaire
